# Hand grip strength in venous thromboembolism: risk of recurrence and mortality

**DOI:** 10.1016/j.rpth.2023.102138

**Published:** 2023-06-29

**Authors:** Oda G.R. Leknessund, Vania M. Morelli, John-Bjarne Hansen, Sigrid K. Brækkan

**Affiliations:** 1 Thrombosis Research Group, Department of Clinical Medicine, UiT - The Arctic University of Norway, Tromsø, Norway; 2 Division of Internal Medicine, University Hospital of North Norway, Tromsø, Norway

**Keywords:** hand strength, recurrence, risk factor, venous thromboembolism, venous thrombosis

## Abstract

**Background:**

There is limited information on the relationship between muscle strength and recurrence and mortality after incident venous thromboembolism (VTE).

**Objectives:**

To investigate whether weak hand grip strength (HGS) was associated with risk of recurrence and mortality in patients with VTE recruited from the general population.

**Methods:**

Participants from the Tromsø Study with a first-time VTE (*n* = 545) were included, and all VTE recurrences and deaths among the participants were recorded in the period 1994 to 2020. Weak HGS was defined as lowest 25th percentile of the general population, and incidence rates for VTE recurrence and mortality according to weak vs normal (>25th percentile) HGS, with 95% CIs, were estimated.

**Results:**

There were 90 recurrences and 350 deaths during a median of 3.7 years of follow-up. The fully adjusted hazard ratio (HR) for overall VTE recurrence for those with weak HGS vs those with normal HGS was 2.02 (95% CI, 1.23-3.30). The corresponding HRs for recurrence were 2.22 (95% CI, 1.18-4.17) in patients with a first deep vein thrombosis and 1.60 (95% CI, 0.72-3.57) in patients with a first pulmonary embolism. The cumulative 1-year survival was 74.9% and 77.8% in those with weak and normal HGS, respectively. For overall mortality after incident VTE, the fully adjusted HR for those with weak HGS was 1.34 (95% CI, 1.04-1.72).

**Conclusion:**

Weak HGS was associated with an increased risk of recurrent VTE, and the association appeared to be particularly pronounced after incident deep vein thrombosis. There was a slightly lower survival probability among those with weak HGS than among those with normal HGS.

## Essentials

• Hand grip strength (HGS) is related to adverse health outcomes and mortality.
• We assessed the association of HGS with recurrent venous thromboembolism and mortality.
• Individuals with weak HGS had a higher risk of venous thromboembolism recurrence compared with those with normal HGS.
• Individuals with weak HGS had a slightly higher mortality risk than those with normal HGS.

## 1 Introduction

Venous thromboembolism (VTE) is a multifactorial disease consisting of deep vein thrombosis (DVT) and pulmonary embolism (PE). VTE is associated with premature mortality, with a 1-year mortality rate of 20% to 25% [1,2] and a high risk of recurrence [3]. The disease is fairly common, as approximately 1 in 12 experience a VTE during their lifetime [4,5]. Furthermore, 30% of the patients with VTE experience recurrent thromboembolic events within 10 years after the incident event [6]. Secondary prevention of VTE with anticoagulants is a 2-edged sword, as extended anticoagulant treatment increases the risk of bleeding [7,8], whereas lack of prevention might lead to VTE recurrence [[9], [10], [11]]. Therefore, identification of risk factors for recurrence and mortality, which can guide classification of patients with a positive benefit-to-harm ratio for extended anticoagulant treatment, is important to reduce the disease burden of VTE.

Muscular strength and physical capability weaken with increasing age [12]. Loss of overall muscle strength, especially lower-extremity strength, may contribute to reduced effectiveness of the muscle vein pump, resulting in altered blood flow and vascular stasis, which is suggested to be of importance in the pathophysiology of VTE development [13]. In a recent study, Houghton et al. [14] reported that reduced calf muscle pump function was associated with increased risk of VTE, particularly DVT formation. Hand grip strength (HGS) is widely used in clinical practice to determine muscle strength and has been reported to serve as a reliable surrogate for more complicated measurement techniques for upper- and lower-extremity strength [15,16]. A weak HGS is associated with a wide range of diseases, including cardiovascular and respiratory diseases, cancer, and mortality [[17], [18], [19], [20], [21]]. In a cohort of 13,704 participants from the Tromsø Study, we found that a weak HGS was associated with a 27% increased risk of first-lifetime VTE [22].

To our knowledge, the relationship between muscle strength and risk of recurrent VTE and mortality has not been previously explored. The aim of this study was, therefore, to investigate the association between HGS and risk of recurrence and mortality in patients with a first VTE. We hypothesized that a weak HGS was associated with higher risk of recurrent VTE and mortality.

## 2 Methods

### 2.1 Study population

The source cohort consisted of 13,704 participants from the surveys 4 to 7 of the Tromsø Study (conducted in 1994-1995, 2001-2002, 2007-2008, and 2015-2016, respectively), a general population cohort with repeated measurements of the inhabitants of Tromsø, Norway [22]. The Tromsø Study surveys 4 to 7 had a participation rate of 65% to 79% of the eligible population. The process of study inclusion is described in Supplementary Figure 1. In brief, all Tromsø Study participants with recorded HGS measurements and no recorded prebaseline VTE event were included in the source cohort. From this cohort, all cases of incident VTE during the period 1994 to 2020 were identified (*n* = 545). Written informed consent to participate in the Tromsø study was obtained from all included participants.

The VTE events were carefully validated by review of medical records, and all cases with symptomatic DVT and/or PE objectively confirmed by radiological procedures were included, as previously described in detail [23,24]. All VTEs were classified as DVT or PE, and cases with concurrent disease were classified as PE. VTE events occurring under the presence of known provoking factors were classified as provoked events, and events occurring under no influence of provoking factors were classified as unprovoked. Provoking factors included surgery or trauma within 8 weeks prior to the event, acute medical conditions (acute myocardial infarction, ischemic stroke, and major infectious disease), active cancer, immobilization (bed rest >3 days, wheelchair use, long-distance travel exceeding 4 hours within the last 14 days prior to the event), and other factors specifically described as provoking by physicians in the medical records, for example, intravascular catheters. A VTE event was classified as cancer-related if the VTE occurred in a patient with an active cancer diagnosis at the time of the event. The incident VTE cohort (*n* = 545) was followed, and all VTE recurrences and deaths among the study participants were recorded until the end of follow-up (December 31, 2020).

### 2.2 Outcome assessment

All recurrent VTE events during follow-up were identified using the same procedure as previously described for the incident events [22]. In brief, the hospital discharge registry and the radiology procedure registry were searched to identify the cases, and medical records were thoroughly reviewed for adjudication and recording of symptomatic, objectively confirmed recurrent VTE events. The VTE registry is a regional registry, and patients who moved outside the catchment area of the hospital were, therefore, censored at the date of migration in the recurrence analyses (*n* = 6). Data on mortality were obtained from the Norwegian Population Registry using the unique national person identification number. This is a national registry and information about mortality could therefore be obtained even for those participants who migrated from Tromsø to other places in the country during follow-up.

### 2.3 Hand grip strength

At inclusion in the Tromsø 4 to 7 surveys, HGS was assessed by trained health care professionals using a standardized study protocol. In surveys 4 to 6 (Tromsø4 to Tromsø6), the Martin Vigorimeter was applied, and this instrument was replaced with the Jamar Digital Dynamometer in Tromsø7. For the Martin Vigorimeter, each participant was allowed 2 attempts with the nondominant hand, and the results were measured in bar units. The highest score out of the 2 attempts was used in the analyses. In survey 7 (Tromsø7), using the Jamar Digital Dynamometer, each participant was allowed 3 attempts for each hand. The highest score out of the 2 first attempts of the nondominant hand was used in the statistical analyses to ensure similarity between surveys 4 to 6 and survey 7.

Participants of the source population were categorized into dynamometer-specific quartiles (as different dynamometers were used in Tromsø4 to Tromsø6 and Tromsø7), according to measures of HGS. The original quartile cut-offs, derived from the source cohort (*n* = 13,704), were used to classify the individuals in the VTE cohort as weak (≤25th percentile) or normal (>25th percentile) HGS. The weak quartile cut-off was chosen on the basis of our previous findings in the Tromsø Study, in which we observed a threshold effect for risk of incidence of VTE according to the lowest quartile of HGS [22].

### 2.4 Other measurements

Baseline information was obtained by physical examinations and comprehensive questionnaires (self-report) at inclusion in the Tromsø Study, and data from the most recent survey preceding the incident VTE event were used. Height and weight were measured with participants wearing light clothes and no shoes, and body mass index (BMI; kg/m^2^) was calculated from these measures. Information on comorbidities was collected via standardized questionnaires, and information about cancer diagnoses was obtained from the Cancer Registry of Norway. Cardiovascular disease (CVD) was classified as a self-reported history of ischemic stroke, myocardial infarction, or angina pectoris. Information on planned duration of anticoagulants was obtained from hospital medical records. In participants with missing information on anticoagulant duration (*n* = 43), the type of incident VTE event was used to determine the anticoagulant duration in accordance with the institutional recommendations during the study period. Provoked DVT events were assigned 3 months of treatment, unprovoked DVT and provoked PE were assigned 6 months of treatment, unprovoked PE was assigned 12 months of treatment, and cancer-related VTE events were assigned more than 1 year of anticoagulant treatment.

### 2.5 Statistical analysis

For the analyses of VTE recurrence, person-time of follow-up was accrued from the date of the first VTE event until VTE recurrence, death, migration, or end of follow-up, whichever came first. The analysis set-up was identical for the mortality analyses, except that recurrent VTE and migration were not included as censoring events. We used the HGS measurement most proximate to the incident VTE event in the analysis. Statistical analyses were performed with STATA 17.0 (Stata Corporation). In cases in which incident VTE and death occurred simultaneously (*n* = 14), 1 day of follow-up was modeled for the mortality analyses.

Crude recurrence rates were computed by dividing the number of events by the total accrued person-time at risk and expressed per 100 person-years. Hazard ratios (HRs) with 95% CIs according to weak (≤25th percentile) vs normal (>25th percentile, reference) HGS were estimated in Cox proportional hazard regression models, with and without adjustment for potentially relevant confounders. Adjustment model 1 included age at index VTE and sex. Model 2 was additionally adjusted for BMI and body height recorded at cohort baseline, and in model 3, we further added adjustments for relevant comorbidities (active cancer, assessed at time of diagnosis of first VTE and previous CVD, assessed at cohort baseline). Since use of anticoagulant treatment might alter the risk of recurrent VTE, we also conducted the analysis adjusting for planned duration of anticoagulation. There were no missing values on age and sex, whereas the variables BMI, body height, cancer, and CVD all had <4% missing data across all quartiles. We also estimated HRs for recurrence and mortality in subgroups of the index VTE event (ie, DVT, PE, and provoked and unprovoked VTE). The proportional hazards assumption was evaluated and verified on the basis of Schoenfeld residuals and graphical inspection of the log-log survival curves and found not violated.

The 1-, 3-, 5-, and 10-year cumulative risks of recurrence and mortality according to HGS were estimated and illustrated with the Kaplan–Meier (KM) failure function (1-KM) and the KM survival function, respectively.

Previous studies have shown a higher recurrence risk among men [[25], [26], [27], [28]], and since HGS is also known to be higher in men than in women, we additionally performed sex-specific analyses using sex-specific quartiles. We also chose to perform sensitivity analysis, in which we excluded those with a cancer-related index VTE as these patients are at even higher risk of recurrence and mortality than patients with noncancer VTE [27,29,30]. Finally, since the risk of recurrence might be overestimated when the risk of mortality is high and differs between exposure groups [31], we accounted for competing risk by death by estimating cumulative incidence functions (Fine and Gray method) and subdistribution HRs with adjustment model 3.

## 3 Results

Study population characteristics and clinical presentation are shown in Table 1. The average age at VTE was higher among participants with a weak HGS (mean, 78.0 years) compared to participants with a normal HGS (mean, 72.3 years). The proportion of women was higher in the weak HGS quartile, and correspondingly, the average body height was lower in this quartile. The prevalence of a history of CVD was highest among those with weak HGS (26.6% vs 20.5%), and the prevalence of active cancer disease at the time of VTE was highest among those with normal HGS (22.3% vs 27.7%). There was no substantial difference regarding the duration of anticoagulant treatment between those with weak and normal HGSs. Detailed information on the clinical presentation of the incident VTE event is stated in Table 1. In brief, there was a slightly higher proportion of DVT than PE and provoked VTE than unprovoked VTE. The type of first VTE was related to the type of recurrence, as patients with a first DVT were more likely to have a second DVT rather than PE and patients with a first PE were more likely to have a second PE (data not shown). The most frequent provoking factors were active cancer (22.3% and 27.7%), immobilization (23.4% and 19.9%), and surgery (15.2% and 16.1%) in weak and normal HGS groups, respectively. Population characteristics and clinical presentations across all 4 quartiles of HGS are shown in Supplementary Table 1.

**Table 1:** Baseline and clinical characteristics of included incident VTE cases (*n* = 545) from the Tromsø Study from 1994 to 2016.

Variables	HGS ≤ 25th percentile (*n* = 184)	HGS > 25th percentile (*n* = 361)
Age, mean ± SD^a^	78.0 ± 7.1	72.3 ± 9.1
Sex, women, % (n)	76.6 (141)	43.8 (158)
BMI (kg/m^2^), mean ± SD	27.4 ± 4.8	27.6 ± 3.9
Height (cm), mean ± SD	163 ± 8.1	171 ± 9.4
History of CVD, % (n)	26.6 (49)	20.5 (74)
Clinical presentation		
Deep vein thrombosis, % (n)	56.5 (104)	52.9 (191)
Pulmonary embolism, % (n)	43.5 (80)	47.1 (170)
Unprovoked, % (n)	44.0 (81)	38.5 (139)
Provoked, % (n)	56.0 (103)	61.5 (222)
Assigned treatment duration with AC (mo), % (n)		
0-3	26.6 (49)	31.6 (114)
3-6	40.2 (74)	35.5 (128)
6-12	16.3 (30)	14.1 (51)
>12	16.9 (31)	18.8 (68)
Provoking factors		
Active cancer, % (n)^b^	22.3 (41)	27.7 (100)
Surgery, % (n)	15.2 (28)	16.1 (58)
Trauma, % (n)	11.4 (21)	6.7 (24)
Acute medical condition, % (n)	10.3 (19)	13.3 (48)
Immobilization, % (n)	23.4 (43)	19.9 (72)
Other provoking factor, % (n)	3.8 (7)	5.6 (20)

AC, anticoagulants; BMI, body mass index; CVD, cardiovascular disease (history of cerebral stroke, myocardial infarction, or angina pectoris at cohort baseline); HGS, hand grip strength; VTE, venous thromboembolism.

a Age at incident VTE event.

b Active cancer diagnosis at time of incident VTE.

### 3.1 Recurrence

**Figure 1:**
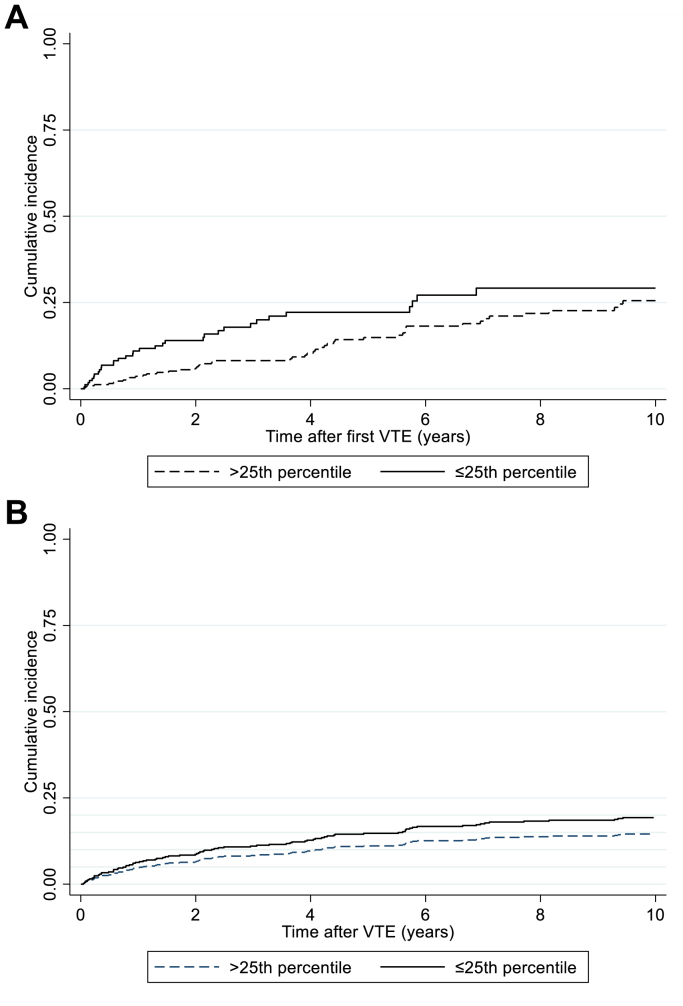
Cumulative incidence of venous thromboembolism (VTE) recurrence according to measures of hand grip strength (≤25th percentile vs >25th percentile). (A) 1-Kaplan-Meier curves. (B) Cumulative incidence after taking competing risk of death into account.

Among the 545 people with incident VTE, 90 experienced a recurrent event during a median of 2.7 years of follow-up. Six participants (*n* = 6) migrated during follow-up and were, therefore, censored at the date of migration in the recurrence analyses. At 1 year, the cumulative incidence of recurrence was 11.0% (95% CI, 7.0-17.1) in those with weak HGS and 3.6% (95% CI, 0.2-6.4) in those with normal HGS (Figure 1A). The corresponding numbers were 20.0% (95% CI, 14.0-28.1) and 8.2% (95% CI, 5.4-12.2) at 3 years and 22.2% (95% CI, 15.8-30.7) and 14.9% (95% CI, 10.7-20.4) at 5 years. The 10-year cumulative incidence was 29.2% (95% CI, 20.9-39.8) in those with weak HGS and 26.6% (95% CI, 20.3-34.3) in those with normal HGS (Figure 1A). The cumulative recurrence incidence remained higher among those with weak HGS than those with normal HGS after taking competing risk of death into account (Figure 1B).

The relative risk of VTE recurrence was notably elevated in those with weak HGS across all adjustment models (model 1: HR, 1.80; 95% CI, 1.12-2.91; model 2: HR, 1.99; 95% CI, 1.22-3.24; model 3: HR, 2.02; 95% CI, 1.23-3.30) (Table 2). The risk estimate was only slightly attenuated after taking competing risk of death into account in the multivariable model (subdistribution HR, 1.86; 95% CI, 1.16-3.00). Further adjustment for planned duration of anticoagulant therapy did not affect the results (data not shown).

**Table 2:** RRs and HRs with 95% CIs of VTE recurrence for hand grip strength ≤25th percentile compared to hand grip strength >25th percentile.

VTE recurrence by percentile groups	Person-years	Events	Crude RR^a^ (95% CI)	Model 1 HR (95% CI)	Model 2 HR (95% CI)	Model 3 HR (95% CI)	Model 3 SHR^b^ (95% CI)
Overall recurrence							
>25th percentile	2012	55	2.73 (2.10-3.56)	1	1	1	1
≤25th percentile	723	35	4.84 (3.48-6.74)	1.80 (1.12-2.91)	1.99 (1.22-3.24)	2.02 (1.23-3.30)	1.86 (1.16-3.00)
Recurrence after provoked							
>25th percentile	1031	28	2.71 (1.87-3.93)	1	1	1	1
≤25th percentile	297	16	5.38 (3.30-8.79)	2.31 (1.14-4.66)	2.60 (1.22-5.51)	2.59 (1.21-5.52)	2.19 (1.07-4.45)
Recurrence after unprovoked							
>25th percentile	980	27	2.75 (1.89-4.02)	1	1	1	1
≤25th percentile	426	19	4.46 (2.85-7.00)	1.42 (0.73-2.78)	1.56 (0.79-3.08)	1.54 (0.77-3.06)	1.56 (0.79-3.08)
Recurrence after PE							
>25th percentile	850	24	2.82 (1.89-4.21)	1	1	1	1
≤25th percentile	314	11	3.50 (1.94-6.32)	1.47 (0.67-3.21)	1.65 (0.74-3.68)	1.60 (0.72-3.57)	1.27 (0.57-2.82)
Recurrence after DVT							
>25th percentile	1161	31	2.67 (1.88-3.80)	1	1	1	1
≤25th percentile	408	24	5.88 (3.94-8.77)	1.97 (1.07-3.65)	2.20 (1.17-4.13)	2.22 (1.18-4.17)	2.24 (1.19-4.21)

Model 1: adjusted for age (at incident VTE) and sex.

Model 2: adjusted for age, sex, BMI, and height.

Model 3: adjusted for age, sex, BMI, height, cardiovascular disease (at cohort baseline), and active cancer (at time of VTE diagnosis).

BMI, body mass index; DVT, deep vein thrombosis; HR, hazard ratio; PE, pulmonary embolism; RR, recurrence rate; SHR, subdistribution HR; VTE, venous thromboembolism.

a Recurrence rate per 100 person-years.

b Denotes the HR after considering a competing risk by death.

Subgroup analyses based on the different clinical presentations of the index event showed a fully adjusted HR for recurrence of 2.59 (95% CI, 1.21-5.52) after incident provoked VTE and 1.54 (95% CI, 0.77-3.06) after incident unprovoked VTE in those with weak vs normal HGSs. The HRs for recurrence after incident DVT and PE were 2.22 (95% CI, 1.18-4.17) and 1.60 (95% CI, 0.72-3.57), respectively.

Several sensitivity analyses were performed to explore the robustness of our results. The results obtained in analyses based on sex-specific quartiles were similar to those obtained in the main analyses (Supplementary Table 2). Furthermore, in analyses restricted to those without cancer-related VTE, the risk of recurrence remained higher in those with weak vs normal HGS both after overall VTE (model 3: HR, 1.90; 95% CI, 1.12-3.20) and provoked index VTE (model 3: HR, 2.23; 95% CI, 0.89-5.57) (Supplementary Table 3).

### 3.2 All-cause mortality

**Figure 2:**
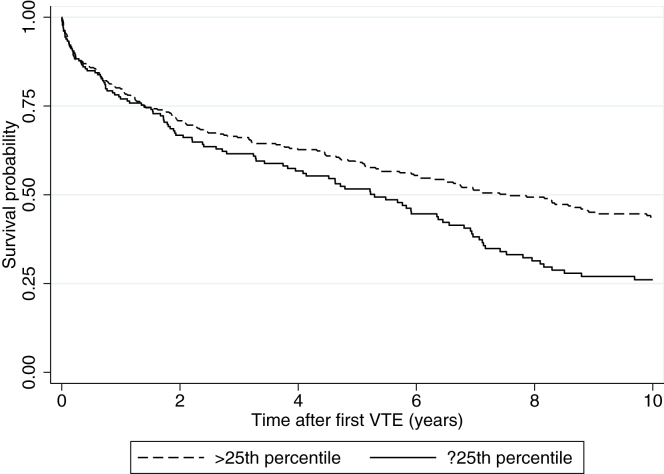
Kaplan-Meier survival probability after venous thromboembolism (VTE) according to measures of hand grip strength (≤25th percentile vs >25th percentile).

A total of 350 deaths occurred during a median of 3.7 years of follow-up from first-time VTE until death or study end. The overall mortality rate was 15.1 (95% CI, 12.8-18.0) per 100 person-years in those with weak HGS and 9.4 (95% CI, 8.2-10.7) per 100 person-years in those with normal HGS (Table 3). The 10-year crude survival probability among participants with a weak and normal HGSs is visualized in Figure 2. The 1-, 3-, and 10-year cumulative survival were 74.9% (95% CI, 68.0-80.6), 60.0% (95% CI, 52.2-66.7), and 25.4% (95% CI, 18.3-33.0) in individuals with weak HGS and 77.8% (95% CI, 73.2-81.8), 64.5% (95% CI, 59.2-69.2), and 42.1% (95% CI, 36.4-47.7) in individuals with normal grip strength, respectively (Figure 2). Correspondingly, the fully adjusted HRs for overall as well as 1-, 3-, and 10-year mortality according to weak vs normal HGSs were 1.34 (95% CI, 1.04-1.72), 1.25 (95% CI, 0.81-1.95), 1.15 (95% CI, 0.82-1.63), and 1.24 (95% CI, 0.94-1.63), respectively (Table 3). Sex-specific mortality analyses and analyses restricted to those without cancer-related index VTE yielded similar results (Supplementary Tables 4 and 5).

**Table 3:** All-cause MRs after incident VTE with 95% CIs for hand grip strength ≤25th percentile compared to hand grip strength >25th percentile.

Mortality by percentile groups	Person-years	Events	Crude MR^a^ (95% CI)	Model 1	Model 2	Model 3
Overall mortality						
>25th percentile	2341	219	9.4 (8.2-10.7)	1	1	1
≤25th percentile	865	131	15.1 (12.8-18.0)	1.23 (0.96-1.56)	1.20 (0.94-1.54)	1.34 (1.04-1.72)
One-year mortality						
>25th percentile	304	71	23.4 (18.5-29.5)	1	1	1
≤25th percentile	153	41	26.8 (19.8-36.4)	1.08 (0.70-1.64)	1.05 (0.68-1.62)	1.25 (0.81-1.95)
Three-year mortality						
>25th percentile	810	116	14.3 (11.9-17.2)	1	1	1
≤25th percentile	401	66	16.5 (12.9-21.0)	1.03 (0.74-1.44)	1.00 (0.71-1.40)	1.15 (0.82-1.63)
Ten-year mortality						
>25th percentile	2156	176	8.2 (7.0-9.5)	1	1	1
≤25th percentile	811	111	13.7 (11.4-16.5)	1.13 (0.87-1.46)	1.09 (0.48-1.43)	1.24 (0.94-1.63)

Model 1: adjusted for age (at incident VTE) and sex.

Model 2: adjusted for age, sex, BMI, and height.

Model 3: adjusted for age, sex, BMI, height, cardiovascular disease (recorded at cohort baseline), and active cancer (recorded at the time of VTE diagnosis).

BMI, body mass index; MR, mortality rate; VTE, venous thromboembolism.

a Mortality rate per 100 person-years.

## 4 Discussion

In this study, we investigated the association between HGS and risk of VTE recurrence as well as the association between HGS and mortality after an incident VTE event. We found that weak HGS, defined as the lowest 25th percentile of the general population, was associated with an approximately 2-fold increased risk of recurrent VTE. The association between HGS and VTE recurrence appeared to be especially pronounced after an index DVT. Comparable results were found in the sex-specific analyses, and the association remained when taking competing risk by death into account and in analyses restricted to individuals without cancer-related VTE. Analyses on mortality revealed a 34% increased risk of mortality in those with weak than normal HGS after an incident VTE event.

To our knowledge, the association between HGS and VTE recurrence has not been explored previously. In a former study, we explored the association between HGS and risk of incident VTE among 13,704 individuals recruited from the Tromsø Study and found that weak HGS was associated with a 27% higher risk of VTE, in particular DVT and unprovoked VTE, with a 52% and 35% increased risk, respectively [22]. Our results were supported by a case-control study of 401 cases and 431 controls reporting that a weak HGS (defined as <15th percentile and lowest tertile) was associated with a 2-fold risk of VTE [32]. On the contrary, Kunutsor et al. [33] found no association between HGS and risk of incident VTE in a cohort of 864 participants from the Kuopio Ischemic Heart Disease Risk Factor Study.

Apparently, a weak HGS was a stronger risk marker for recurrent events than for incident VTE events, both in terms of relative risk and absolute risk difference. It is well established that risk factors may have differential effects on first and recurrent VTE events [34,35]. For example, age is a strong risk factor for a first-time event but not associated with recurrence risk [36]. Further, the lifetime risk of a first VTE event is equal for both sexes [4,5], whereas several studies have reported a higher recurrence risk in men than women [28]. A potential explanation for this phenomenon is “index event bias” or “collider bias,” which occurs when study participants are selected based on the presence of a specific disease [37].

We found that the risk of recurrence according to weak HGS was particularly high after a first DVT. HGS is commonly used as a proxy for overall muscle strength, and it correlates well with lower leg strength [15,16,38]. Weak muscle strength in the lower extremities affects the venous circulation through reduced calf muscle pump function resulting in disturbed blood flow and stasis, which increases the risk of VTE [14]. After an incident VTE, residual vein obstruction as well as local damage of the veins and vascular valves may cause sustained inflammation and valvular reflux and thereby increase the risk of recurrence [[39], [40], [41], [42], [43]]. Considering that HGS appeared to be more strongly associated with recurrent than with incident VTE, particularly recurrence after DVT, it might be speculated that the combination of weak calf muscles and pathophysiological changes in the veins following an incident event interacted to yield a synergistic effect on recurrence risk. Intravascular pathophysiological changes in the peripheral deep veins can be found in less than half of patients with PE [44], which may further explain the observed higher recurrence risk after DVT than after PE in those with low HGS. Unfortunately, we did not have information on the exact location of the incident DVT and if the recurrent DVT was ipsilateral or contralateral. An increased risk of ipsilateral DVT after a first incident DVT could support the hypothesis that pathologic damage of the endothelium and valves, in combination with weak calf muscles, increases the risk of VTE recurrence.

We observed that weak HGS was associated with a higher risk of VTE recurrence after a first provoked VTE. This observation could not be explained by active cancer, as exclusion of cancer-related events only slightly attenuated the association. Potentially, damage to veins and vascular valves following surgery or trauma could lead to higher recurrence risk in those with poor calf muscle strength. However, the association between provoked VTE and recurrence remained after exclusion of this patient group in further sensitivity analyses. A weak HGS is often used as a marker of frailty [[45], [46], [47]]. Thus, it could be that underlying diseases and conditions leading to provoked VTE in frail individuals (eg, in the setting of hospitalization for medical conditions with concomitant immobilization) also contribute to increase in the recurrence risk. We performed several sensitivity analyses and adjustments, attempting to account for potential confounding by comorbidities and frailty. We adjusted for important risk factors (ie, age, sex, CVD, cancer, BMI, and height) in our multivariable model. We also performed sensitivity analyses where participants with cancer-related index VTE were excluded (Supplementary Table 3). Unfortunately, due to the limited size of the present study and lack of detailed information on multimorbidity, we could not explore this relationship further in subgroups. Furthermore, since index event bias cannot be ruled out, causal inference of our findings should be interpreted with caution.

HGS is a simple measure associated with overall muscle strength [15,16] and could be easily used, for example, in general practice. HGS as a stand-alone test to evaluate risk of recurrent VTE would probably be of limited practical use, but it could possibly be used as a part of a recurrence risk prediction model. Further studies are needed to address the predictive capability of HGS in combination with other risk markers.

Patients with VTE have a substantially higher risk of mortality than the general population [27,48,49]. We found that patients with VTE with a weak HGS had a 34% overall increased risk of mortality compared with those with normal HGS. Although our study is the first to assess the association between HGS and mortality in patients with VTE, the association has been extensively explored in other populations. A systematic review and meta-analysis conducted in 2010, including 23 studies on the association between HGS and all-cause mortality in community-dwelling populations, reported that weak HGS (<25th percentile) was associated with a 67% (HR, 1.67; 95% CI, 1.45-1.93) higher risk of all-cause mortality [50], a finding that is also supported by later publications [17,[51], [52], [53]]. The low relative increase in mortality risk according to HGS observed in patients with VTE is likely explained by the higher baseline mortality risk in this patient group as well as index event bias, old age, and higher prevalence of comorbidities [18,46,51,52].

Strengths of this study include the prospective cohort design with unselected patients with VTE recruited from the general population with a relatively long follow-up time. The participation rate in the Tromsø Study was high, and health care and relevant diagnostics for VTE were solely provided by one regional hospital, which secures a comprehensive and accurate outcome detection. All incident and recurrent VTEs were carefully adjudicated from hospital records using the same standard procedure. Baseline information was collected via standardized measurements, registries, and validated questionnaires, and we, therefore, have proper information about exposure and important confounders. In addition, we performed several sensitivity analyses and accounted for competing risk by death, which supported the robustness of our results.

The present study has some limitations. HGS was assessed at the regular Tromsø Study surveys and, consequently, measured several years before VTE in a majority of the participants. As HGS could change over time, this might have led to an underestimation of the true association due to random misclassification and regression dilution bias. In addition, several consequences of VTE could influence HGS postevent, such as impaired physical function and decreased mobility, which could further contribute to dilute the results. On the contrary, features associated with a weak HGS, such as frailty and multimorbidity, could potentially confound the observed association between weak HGS and recurrence. Nonetheless, HGS could be a useful marker to assess recurrence risk related to such conditions. Unfortunately, we did not have information on the actual duration of anticoagulant treatment after the incident event. However, adjustment for planned duration of anticoagulants, which is expected to adequately reflect the actual duration in most cases, did not impact our results. Nevertheless, we cannot rule out that frail individuals could be more prone to have their course of anticoagulation abbreviated due to complications or comorbidities. Lastly, information on race and ethnicity among the participants was not collected. The Tromsø Study is a population-based study of the inhabitants of Tromsø, a Norwegian city with relatively few immigrants. The present study population consisted of a predominantly Caucasian population, and the findings might, therefore, not be generalizable across all ethnicities.

In conclusion, weaker HGS was associated with a 2-fold increased risk of recurrent VTE, and the association appeared to be particularly pronounced after an incident DVT. As HGS is merely a proxy for muscle strength in the lower extremities, future studies should explore the possible role of muscle strength and calf muscle pump function as a risk factor for VTE recurrence.
