# Supplementary figures and images for: Hand grip strength in venous thromboembolism: risk of recurrence and mortality
Source: Res Pract Thromb Haemost. 2023 Jun 29;7(5):102138. doi: 10.1016/j.rpth.2023.102138 (PMC10439395; doi:10.1016/j.rpth.2023.102138)

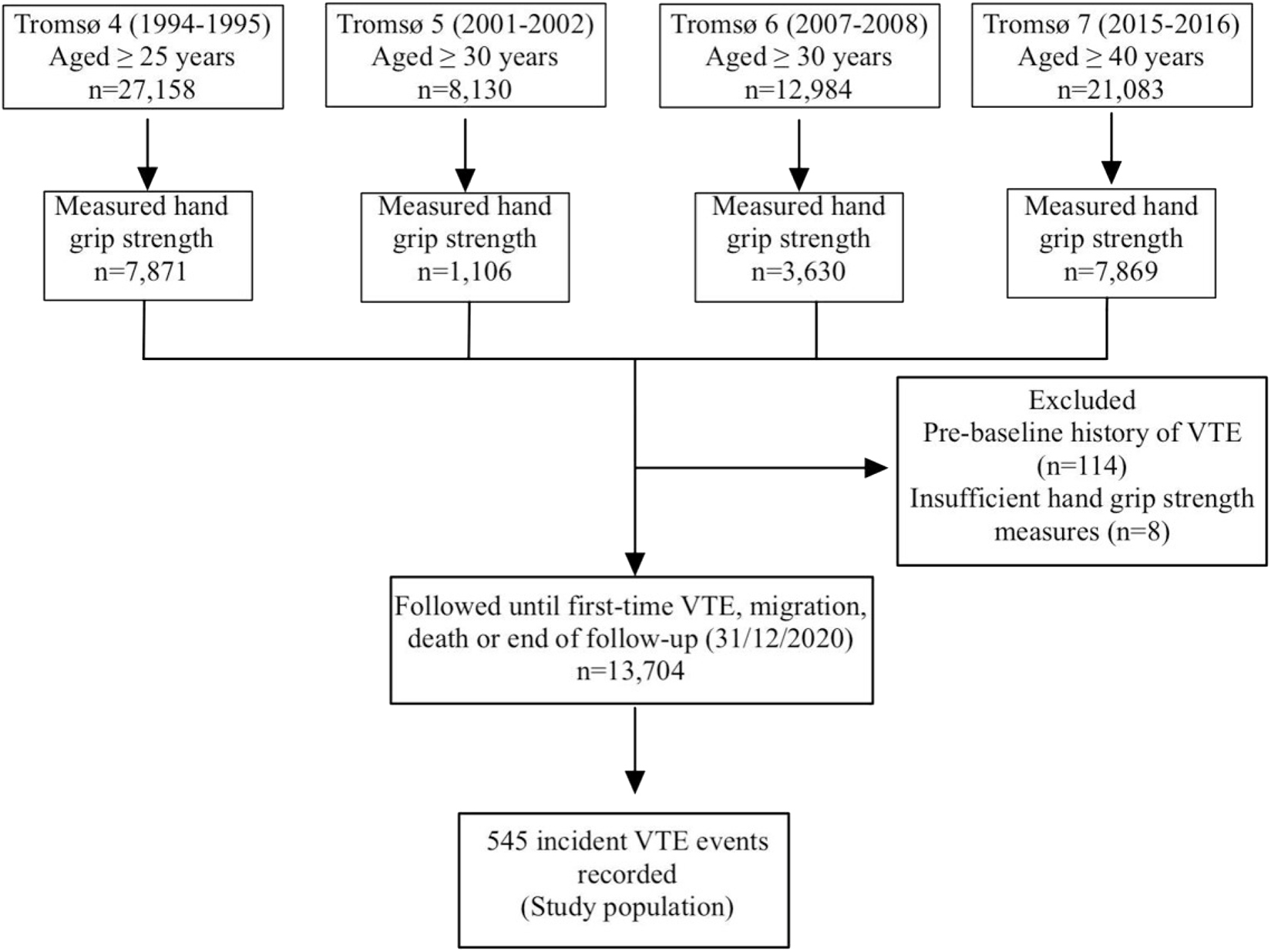

Supplement: Supplementary Figure 1 [file figs1.jpg]
