# Supplementary material for: Hand grip strength in venous thromboembolism: risk of recurrence and mortality
Source: Res Pract Thromb Haemost. 2023 Jun 29;7(5):102138. doi: 10.1016/j.rpth.2023.102138 (PMC10439395; doi:10.1016/j.rpth.2023.102138)
Supplement: Supplementary Material — 1 [file mmc1.docx]

**Supplementary material**

**Supplementary figure 1:** Overview of included and excluded participants from Tromsø4-Tromsø7 (1994-2016).

**
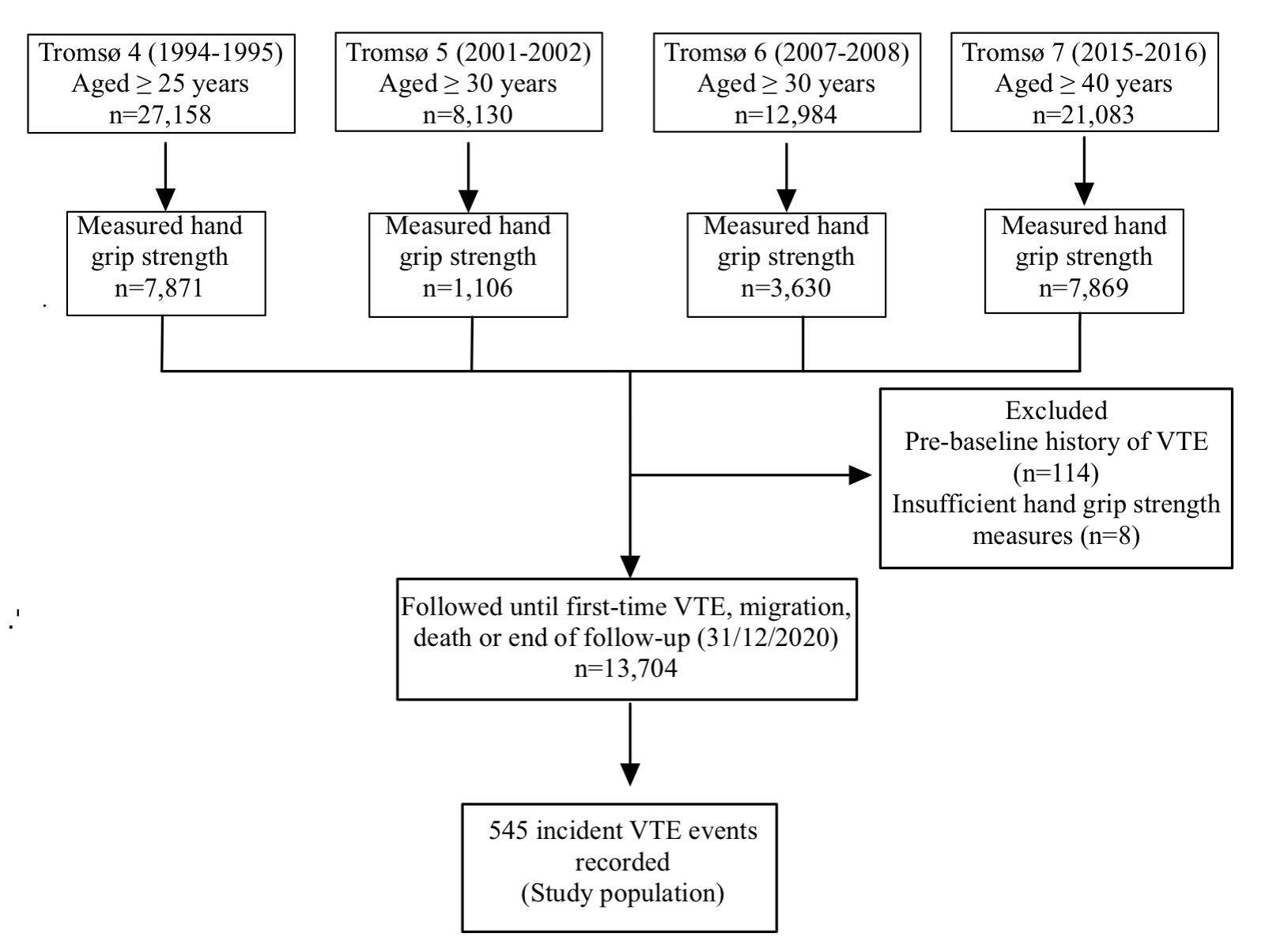
**

**Supplementary table 1:** Baseline and clinical characteristics of included incident venous thromboembolism cases (n= 545) across quartiles of hand grip strength, The Tromsø Study 1994-2016

| **Variables** | **Quartile 1 (n=184)** | **Quartile 2 (n=133)** | **Quartile 3 (n=124)** | **Quartile 4 (n=104)** |
| --- | --- | --- | --- | --- |
| Age, mean ± SD * | 78.0 ± 7.1 | 75.1 ± 9.3 | 73.1 ± 7.3 | 68.0 ± 9.3 |
| Sex, women, % (n) | 76.6 (141) | 59.4 (79) | 37.9 (47) | 30.8 (32) |
| BMI (kg/m2), mean ± SD | 27.4 ± 4.8 | 27.4 ± 4.0 | 27.3 ± 3.6 | 28.2 ± 4.2 |
| Height (cm), mean ± SD | 163 ± 8.1 | 167 ± 7.9 | 171 ± 9.4 | 175 ± 9.7 |
| History of CVD, % (n) | 26.6 (49) | 21.8 (29) | 17.7 (22) | 22.1 (23) |
| **Clinical presentation** |  |  |  |  |
| Deep vein thrombosis, % (n) | 56.5 (104) | 52.6 (70) | 52.4 (65) | 53.9 (56) |
| Pulmonary embolism, % (n) | 43.5 (80) | 47.4 (63) | 47.6 (59) | 46.2 (48) |
| Unprovoked, % (n) | 44.0 (81) | 41.4 (55) | 38.7 (48) | 34.6 (36) |
| Provoked, % (n) | 56.0 (103) | 58.7 (78) | 61.3 (76) | 65.4 (68) |
| Treatment duration with AC (months), % (n) |  |  |  |  |
| 0-3 | 26.6 (49) | 32.3 (43) | 30.7 (38) | 31.7 (33) |
| 3-6 | 40.2 (74) | 33.8 (45) | 36.3 (45) | 36.5 (38) |
| 6-12 | 16.3 (30) | 17.3 (23) | 12.9 (16) | 11.5 (12) |
| > 12 | 16.9 (31) | 16.5 (22) | 20.2 (25) | 20.2 (21) |
| **Provoking factors** |  |  |  |  |
| Active cancer, % (n) † | 22.3 (41) | 26.3 (35) | 28.2 (35) | 28.9 (30) |
| Surgery, % (n) | 15.2 (28) | 14.4 (19) | 14.5 (18) | 20.2 (21) |
| Trauma, % (n) | 11.4 (21) | 10.6 (14) | 3.2 (4) | 5.8 (6) |
| Acute medical condition, % (n) | 10.3 (19) | 13.6 (18) | 12.9 (16) | 13.5 (14) |
| Immobilization, % (n) | 23.4 (43) | 18.1 (24) | 21.0 (26) | 21.2 (22) |
| Other provoking factor, % (n) | 3.8 (7) | 3.8 (5) | 7.3 (9) | 5.8 (6) |

BMI – body mass index, SD – Standard deviation, CVD – cardiovascular disease (history of cerebral stroke, myocardial infarction or angina pectoris recorded at cohort baseline), AC – anticoagulants

*Age at incident VTE event

† Active cancer diagnosis at time of incident VTE

**Supplementary table 2** Sex-specific quartiles. Recurrence rates (RRs) and hazard ratios (HRs) with 95% confidence intervals (CIs) of venous thromboembolism (VTE) recurrence for hand grip strength ≤25th sex-specific percentile compared to hand grip strength >25th sex-specific percentile

|  | **Person years** | **Events** | **Crude RR* (95% CI)** | **HR Model 1** | **HR Model 2** | **HR Model 3** | **SHR†  Model 3** |
| --- | --- | --- | --- | --- | --- | --- | --- |
| **Overall recurrence** | |  |  |  |  |  |  |
| >25^th^ perc | 1987 | 55 | 2.77 (2.13-3.61) | 1 | 1 | 1 | 1 |
| ≤25^th^ perc | 748 | 35 | 4.68 (3.36-6.52) | 1.54 (0.98-2.42) | 1.71 (1.08-2.72) | 1.73 (1.09-2.75) | 1.68 (0.93-3.03) |
| **Recurrence after provoked** | |  |  |  |  |  |  |
| >25^th^ perc | 1016 | 28 | 2.76 (1.90-3.99) | 1 | 1 | 1 | 1 |
| ≤25^th^ perc | 313 | 16 | 5.12 (3.14-8.35) | 1.87 (0.96-3.65) | 2.13 (1.03-4.38) | 2.11 (1.01-4.38) | 1.65 (0.79-3.44) |
| **Recurrence after unprovoked** | |  |  |  |  |  |  |
| >25^th^ perc | 971 | 27 | 2.78 (1.91-4.06) | 1 | 1 | 1 | 1 |
| ≤25^th^ perc | 435 | 19 | 4.36 (2.78-6.84) | 1.33 (0.72-2.46) | 1.45 (0.77-2.74) | 1.43 (0.76-2.71) | 1.31 (0.72-2.39) |
| **Recurrence after PE** | |  |  |  |  |  |  |
| >25^th^ perc | 798 | 22 | 2.76 (1.81-4.19) | 1 | 1 | 1 | 1 |
| ≤25^th^ perc | 366 | 13 | 3.55 (2.06-6.11) | 1.23 (0.60-2.50) | 1.51 (0.71-3.22) | 1.52 (0.71-3.25) | 1.21 (0.59-2.51) |
| **Recurrence after DVT** | |  |  |  |  |  |  |
| >25^th^ perc | 1188 | 33 | 2.78 (1.97-3.91) | 1 | 1 | 1 | 1 |
| ≤25^th^ perc | 381 | 22 | 5.77 (3.80-8.76) | 1.75 (0.98-3.13) | 1.91 (1.05-3.44) | 1.91 (1.05-3.48) | 1.68 (0.93-3.03) |

*Recurrence rate per 100-person-years

† SHR denotes the HR after taking competing risk by death into account

Model 1: Adjusted for age (at incident VTE) and sex

Model 2: Adjusted for age, sex, BMI and height

Model 3: Adjusted for age, sex, BMI, height, CVD (recorded at cohort baseline), active cancer (recorded at time of VTE diagnosis)

**Supplementary table 3** Cancer related VTE events excluded. Recurrencerates (RRs) and hazard ratios (HRs) with 95% confidence intervals (CIs) of venous thromboembolism (VTE) recurrence for hand grip strength ≤25th percentile compared to hand grip strength >25th percentile with first-cancer related VTE events excluded from the analysis.

|  | **Person years** | | **Events** | **Crude RR† (95% CI)** | **HR Model 1** | **HR Model 2** | **HR Model 3** | **SHR‡ Model 3** |
| --- | --- | --- | --- | --- | --- | --- | --- | --- |
| **Overall VTE*** | |  |  |  |  |  |  |  |
| >25^th^ perc | 1837 | | 49 | 2.67 (2.02-3.53) | 1 | 1 | 1 | 1 |
| ≤25^th^ perc | 676 | | 30 | 4.44 (3.10-6.35) | 1.70 (1.02-2.84) | 1.89 (1.12-3.19) | 1.90 (1.12-3.20) | 1.79 (1.08-2.99) |
| **Provoked VTE*** | | |  |  |  |  |  |  |
| >25^th^ perc | 857 | | 22 | 2.57 (1.69-3.90) | 1 | 1 | 1 | 1 |
| ≤25^th^ perc | 250 | | 11 | 4.40 (2.44-7.94) | 1.91 (0.83-4.39) | 2.26 (0.91-5.62) | 2.23 (0.89-5.57) | 1.87 (0.80-4.40) |

* First cancer-related VTE events excluded from the analysis

†Recurrence rate per 100-person-years

‡SHR denotes the HR after taking competing risk by death into account

Model 1: Adjusted for age (at incident VTE) and sex

Model 2: Adjusted for age, sex, BMI and height

Model 3: Adjusted for age, sex, BMI, height, CVD (recorded at cohort baseline)

**Supplementary table 4** Sex-specific quartiles. All-cause mortality rates (MRs) after incident venous thromboembolism with 95% confidence intervals (CIs) per 100 person-years for lowest sex-specific 25^th^ percentile of hand grip strength compared to >25^th^ percentile.

|  | **Person years** | | | **Events** | | | | **Crude MR  (95% CI)** | | | **Model 1** | | **Model 2** | | **Model 3** | |
| --- | --- | --- | --- | --- | --- | --- | --- | --- | --- | --- | --- | --- | --- | --- | --- | --- |
| **Overall mortality** | | |  | |  | | |  | | |  | |  | |  | |
| >25^th^ perc | 2298 | | | 208 | | | | 9.1 (7.9-10.4) | | | 1 | | 1 | | 1 | |
| ≤25^th^ perc | 908 | | | 142 | | | | 15.7 (13.3-18.4) | | | 1.26 (1.00-1.58) | | 1.24 (0.98-1.56) | | 1.35 (1.06-1.72) | |
| **One year mortality** | | | | | | | |  | |  | |  | |  | |  |
| >25^th^ perc | 298 | | | 66 | | | | 22.1 (17.4-28.2) | | | 1 | | 1 | | 1 | |
| ≤25^th^ perc | 158 | | | 46 | | | | 29.1 (21.8-38.8) | | | 1.23 (0.82-1.82) | | 1.19 (0.79-1.80) | | 1.30 (0.85-1.97) | |
| **Three-year mortality** | |  | |  | | | |  | | |  | |  | |  | |
| >25^th^ perc | 804 | | | 108 | | | | 13.4 (11.1-16.2) | | | 1 | | 1 | | 1 | |
| ≤25^th^ perc | 407 | | | 74 | | | | 18.2 (12.5-22.8) | | | 1.19 (0.87-1.63) | | 1.15 (0.84-1.59) | | 1.24 (0.89-1.72) | |
| **Ten-year mortality** | | | | | |  |  | |  | |  | |  | |  | |
| >25^th^ perc | 2128 | | | 169 | | | | 7.9 (6.8-9.2) | | | 1 | | 1 | | 1 | |
| ≤25^th^ perc | 839 | | | 118 | | | | 14.1 (11.7-16.8) | | | 1.19 (0.93-1.52) | | 1.15 (0.89-1.49) | | 1.28 (0.98-1.67) | |

*Mortality rate per 100-person-years

Model 1: Adjusted for age (at incident VTE) and sex

Model 2: Adjusted for age, sex, BMI and height

Model 3: Adjusted for age, sex, BMI, height, CVD (recorded at cohort baseline), active cancer (recorded at time of VTE diagnosis)

**Supplementary table 5** Cancer related events excluded. All-cause mortality rates (MRs) after incident venous thromboembolism with 95% confidence intervals (CIs) per 100 person-years for lowest 25^th^ percentile of hand grip strength compared to >25^th^ percentile with first-cancer related VTE events excluded from the analysis.

|  | **Person years** | | | | **Events** | **Crude MR*  (95% CI)** | **Model 1** | **Model 2** | **Model 3** |
| --- | --- | --- | --- | --- | --- | --- | --- | --- | --- |
| **Overall mortality** | |  | | |  |  |  |  |  |
| >25^th^ perc | 2158 | | | | 129 | 6.0 (5.0-7.1) | 1 | 1 | 1 |
| ≤25^th^ perc | 816 | | | | 94 | 11.5 (9.4-14.1) | 1.39 (1.03-1.87) | 1.36 (1.00-1.84) | 1.36 (1.00-1.84) |
| **One-year mortality** | | |  | |  |  |  |  |  |
| >25^th^ perc | 241 | | | | 19 | 7.9 (5.0-12.4) | 1 | 1 | 1 |
| ≤25^th^ perc | 131 | | | | 14 | 10.7 (6.3-18.0) | 1.00 (0.47-2.11) | 1.00 (0.47-2.12) | 0.94 (0.44-2.01) |
| **Three-year mortality** | | | |  |  |  |  |  |  |
| >25^th^ perc | 688 | | | | 41 | 5.7 (4.4-8.1) | 1 | 1 | 1 |
| ≤25^th^ perc | 360 | | | | 31 | 8.6 (6.1-12.2) | 1.06 (0.64-1.77) | 1.03 (0.61-1.72) | 0.97 (0.58-1.63) |
| **Ten-year mortality** | | | |  |  |  |  |  |  |
| >25^th^ perc | 1981 | | | | 92 | 4.7 (3.8-5.7) | 1 | 1 | 1 |
| ≤25^th^ perc | 762 | | | | 74 | 9.7 (7.7-12.2) | 1.21 (0.86-1.69) | 1.17 (0.83-1.64) | 1.16 (0.82-1.64) |

*Mortality rate per 100-person-years

Model 1: Adjusted for age (at incident VTE) and sex

Model 2: Adjusted for age, sex, BMI and height

Model 3: Adjusted for age, sex, BMI, height, CVD (recorded at cohort baseline)
